# Pyrosequencing-based analysis reveals a novel capsular gene cluster in a KPC-producing *Klebsiella pneumoniae* clinical isolate identified in Brazil

**DOI:** 10.1186/1471-2180-12-173

**Published:** 2012-08-11

**Authors:** Pablo Ivan Pereira Ramos, Renata Cristina Picão, Eliana Carolina Vespero, Marsileni Pelisson, Luiz Fernando Goda Zuleta, Luiz Gonzaga P Almeida, Alexandra L Gerber, Ana Tereza R Vasconcelos, Ana Cristina Gales, Marisa Fabiana Nicolás

**Affiliations:** 1 Laboratório Nacional de Computação Científica (LNCC), Petrópolis, Rio de Janeiro, Brazil; 2 Instituto de Microbiologia Paulo de Góes, Universidade Federal do Rio de Janeiro, Rio de Janeiro, Brazil; 3 Departamento de Patologia Clínica, Análises Clínicas e Toxicologia, Universidade Estadual de Londrina, Londrina, Brazil; 4 Laboratório ALERTA, Divisão de Doenças Infecciosas, Universidade Federal de São Paulo, São Paulo, Brazil

**Keywords:** Capsular gene cluster, Capsular polysaccharide, K-antigen, KPC-producing *K. pneumoniae*, Molecular serotyping, Monosaccharide biosynthesis pathways

## Abstract

**Background:**

An important virulence factor of *Klebsiella pneumoniae* is the production of capsular polysaccharide (CPS), a thick mucus layer that allows for evasion of the host's defense and creates a barrier against antibacterial peptides. CPS production is driven mostly by the expression of genes located in a locus called *cps*, and the resulting structure is used to distinguish between different serotypes (K types). In this study, we report the unique genetic organization of the *cps* cluster from *K. pneumoniae* Kp13, a clinical isolate recovered during a large outbreak of nosocomial infections that occurred in a Brazilian teaching hospital.

**Results:**

A pyrosequencing-based approach showed that the *cps* region of Kp13 (*cps*_Kp13_) is 26.4 kbp in length and contains genes common, although not universal, to other strains, such as the *rml*BADC operon that codes for L-rhamnose synthesis. *cps*_Kp13_ also presents some unique features, like the inversion of the *wzy* gene and a unique repertoire of glycosyltransferases. In silico comparison of *cps*_Kp13_ RFLP pattern with 102 previously published *cps* PCR-RFLP patterns showed that *cps*_Kp13_ is distinct from the C patterns of all other K serotypes. Furthermore, in vitro serotyping showed only a weak reaction with capsular types K9 and K34. We confirm that K9 *cps* shares common genes with *cps*_Kp13_ such as the *rml*BADC operon, but lacks features like *uge* and Kp13-specific glycosyltransferases, while K34 capsules contain three of the five sugars that potentially form the Kp13 CPS.

**Conclusions:**

We report the first description of a *cps* cluster from a Brazilian clinical isolate of a KPC-producing *K. pneumoniae*. The gathered data including K-serotyping support that Kp13’s K-antigen belongs to a novel capsular serotype. The CPS of Kp13 probably includes L-rhamnose and D-galacturonate in its structure, among other residues. Because genes involved in L-rhamnose biosynthesis are absent in humans, this pathway may represent potential targets for the development of antimicrobial agents. Studying the capsular serotypes of clinical isolates is of great importance for further development of vaccines and/or novel therapeutic agents. The distribution of K-types among multidrug-resistant isolates is unknown, but our findings may encourage scientists to perform K-antigen typing of KPC-producing strains worldwide.

## Background

*Klebsiella pneumoniae* is a Gram-negative, rod-shaped bacterium frequently associated with nosocomial and community-acquired infections [1]. Over the past decade, healthcare practitioners have observed the rapid evolution of antimicrobial resistance among *K. pneumoniae* clinical isolates worldwide. The emergence and subsequent global spread of strains producing *Klebsiella pneumoniae* carbapenemase (KPC) represents a significant threat to public health [2]. The gene encoding this β-lactam resistance factor is frequently carried along with genes conferring resistance to multiple classes of antimicrobial agents. As a result, the therapeutic options to treat infections caused by KPC-producing *K. pneumoniae* are generally scarce and in some instances limited to polymyxins [2].

The development of an effective response against *K. pneumoniae* infections depends on the integrity of the immune system. Indeed, many authors have provided evidence that activation of the inflammatory response is required to clear such infections [3-5]. Unfortunately, most patients infected by multidrug-resistant *K. pneumoniae* have serious underlying conditions and/or a compromised immune status [1,6]. Capsule production is believed to be one of the most important virulence factors for this species. The polysaccharide matrix found on its cell surface may prevent desiccation, confer adherence to host cells and protect it against both non-specific and specific host immunity [7]. However, there are differences in the degree of virulence conferred by different *Klebsiella* capsule types, possibly depending on the mannose and/or rhamnose content of the CPS [1]. The *K. pneumoniae* capsule is generally composed of acidic polysaccharides, including uronic acid repeats and, in several instances, mannose, rhamnose, galactose, pyruvate and fucose residues [8]. The genes involved in the biosynthesis, transport and assembly of *K. pneumoniae* and *E. coli* group 1 capsules are found at a locus called *cps*, which is organized similarly in the two species [9]. The biosynthetic process of both types of capsules is also related between the two bacteria. Briefly, CPS synthesis initially takes place on the cytoplasmic side of the inner membrane with the assembly of individual sugar repeat residues which are linked by the sequential activities of specific glycosyltransferases (GTs) [10]. These are then flipped across the inner membrane by the action of the Wzx protein and undergo polymerization by the Wzy protein [11]. Polymerization control and translocation of the nascent polymer to the cell surface occurs with the coordinated action of Wza, Wzb and Wzc proteins [12].

To date, a variety of *cps* gene clusters have been characterized in *Klebsiella* spp., mostly from isolates recovered in the USA, Asia and Europe [13-15]. To our knowledge, there have been no studies on the *cps* organization of *K. pneumoniae* isolates from Brazil, KPC-producing or otherwise. Here, we report the unique *cps* organization of a KPC-producing *K. pneumoniae* isolate showing multidrug resistance. This bacterium was responsible for a large nosocomial outbreak in a teaching hospital located in Southern Brazil (Ana C. Gales, personal communication).

## Results and Discussion

### General features of the *cps*_Kp13_ gene cluster

**Figure 1:**
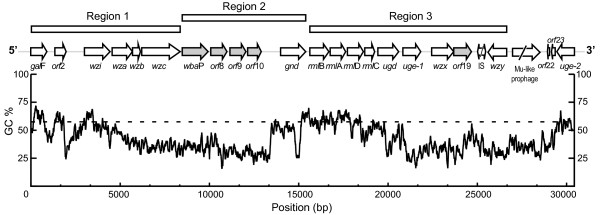
**Overall organization of the *cps* cluster of *K. pneumoniae* Kp13.** The *cps*_Kp13_ spans *gal*F to *wzy*. ORFs are represented by arrows (gray for those encoding glycosyltransferases and double-headed for possible mobile elements). Rectangles above the ORFs represent distinct variably conserved regions of the *cps* cluster as discussed in the text. A plot of the GC content of the region using a 100-bp sliding window is shown below. The dashed horizontal line represents the mean GC content of the entire Kp13 chromosome.

The *cps*_Kp13_ gene cluster is 26.4 kbp in length and contains 20 open reading frames (ORFs) from *gal*F to *wzy* (Figure 1, Table 1). The average GC content of these genes is 42%, which is lower than the average GC content of the entire Kp13 genome (57.5%, data not shown). Comparable GC content has been reported for twelve other *K. pneumoniae cps* clusters [15].

**Table 1:** **General features of the 20 coding sequences identified in the Kp13 *cps* gene cluster**

**ORF**	**Size (bp)**	**%GC**	**Gene name**	**Product**	**EC number**	**Best BLASTP hit (accession number) (identity)**
KP03136	900	59.02	*gal*F	UTP--glucose-1-phosphate uridylyltransferase	2.7.7.9	*K. pneumoniae* strain NK8 (BAI43699) (100%)
KP03135	627	58.41	*orf*2	Uncharacterized phosphatidic acid phosphatase protein	3.1.3.4	*K. pneumoniae* strain MGH 78578 (ABR77932) (100%) and strain VGH404 serotype K5 (BAI43755) (100%).
KP03809	1,431	55.99	*wzi*	Capsule assembly 55.8 kDa protein		*K. pneumoniae* strain VGH484 serotype K9 (BAI43775) (98%)
KP03808	1,131	45.15	*wza*	Capsule polysaccharide export protein		*K. pneumoniae* strain VGH484 serotype K9 (BAI43776) (97%)
KP03807	438	39.69	*wzb*	Protein-tyrosine-phosphatase	3.1.3.48	*K. pneumoniae* strain MGH 78578 (ABR77929) (78%)
KP03806	2,154	35.61	*wzc*	Uncharacterized tyrosine-protein kinase	2.7.10.-	*K. pneumoniae* strain MGH 78578 (ABR77928) (79%)
KP31533	1,446	35.2	*wba*P	Undecaprenolphosphate Gal-1-P transferase	2.-.-.-	*K. pneumoniae* strain MGH 78578 (ABR77927) (79%)
KP03804	906	37.51	*orf*8	Uncharacterized glycosyltransferase family 2	2.4.1-	*K. pneumoniae* strain A1517 (BAF75773) (67%)
KP03803	894	30.99	*orf*9	Uncharacterized glycosyltransferase family 2	2.4.1-	*Dickeya dadantii* (ADM97617) (63%)
KP03802	759	29.79	*orf*10	Uncharacterized glycosyltransferase	2.4.1.-	*D. dadantii* (ADM97619) (57%)
KP31534	1,404	51.46	*gnd*	6-phosphogluconate dehydrogenase, decarboxylating	1.1.1.44	*K. pneumoniae* strain VGH484 serotype K9 (BAI43786) (99%)
KP31530	1,062	59.25	*rml*B	dTDP-D-glucose 4,6-dehydratase	4.2.1.46	*K. pneumoniae* strain VGH484 serotype K9 (BAI43787) (98%)
KP03797	867	58.74	*rml*A	Glucose-1-phosphate thymidylyltransferase	2.7.7.24	*Escherichia coli* HS (EFK17576) (98%)
KP03796	888	61.5	*rml*D	dTDP-4-dehydrorhamnose reductase	1.1.1.133	*K. pneumoniae* strain MGH 78578 (ABR77913) (98%)
KP03795	552	54.41	*rml*C	dTDP-4-dehydrorhamnose 3,5-epimerase	5.1.3.13	*K. pneumoniae* strain VGH484 serotype K9 (BAI43790) (99%)
KP03794	1,164	50.82	*ugd*	UDP-glucose 6-dehydrogenase	1.1.1.22	*K. pneumoniae* strain NK8 (BAI43716) (100%) and strain VGH404 serotype K5 (BAI43755) (100%)
KP03793	999	41.92	*uge*-1	Uridine diphosphate galacturonate 4-epimerase	5.1.3.6	*K. pneumoniae* subsp. *rhinoscleromatis* ATCC 13884 (EEW43608) (97%)
KP31531	1,233	31.57	*wzx*	K-antigen flippase Wzx		*E. coli* TA27 (ZP_07523140) (64%)
KP03791	990	31.32	*orf*19	Uncharacterized glycosyltransferase family 2	2.4.1.-	*Cronobacter sakazakii* (ABX51890) (33%)
KP03789	1,044	29.61	*wzy*	K antigen polymerase Wzy		*Thermoanaerobacter wiegelii* (ACF14522) (35%)

**Figure 2:**
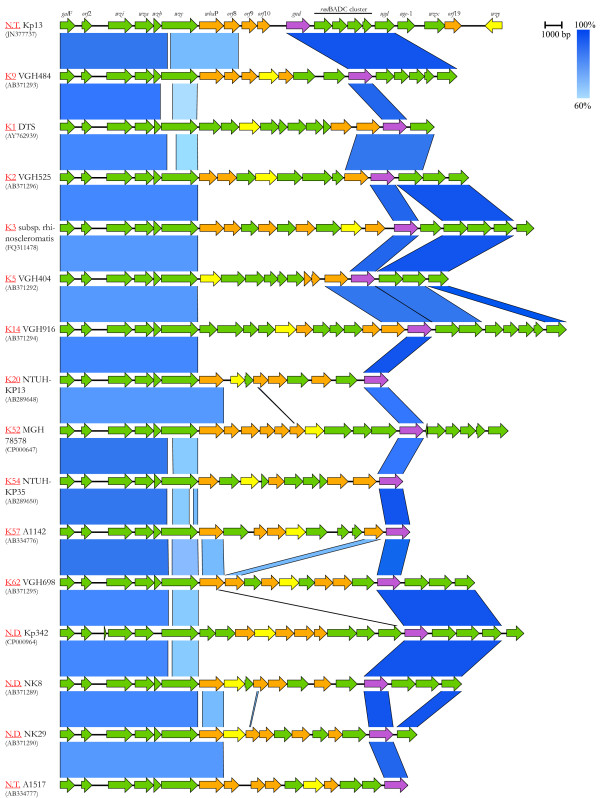
**Comparison of sequenced *K. pneumoniae cps* loci.** For each *cps* cluster, a two-way comparison with the clusters immediately above and/or below is presented. The K-type of each compared cluster is shown in red, followed by the strain/isolate identification and its NCBI accession number in parentheses. The blue segments connecting each cluster represent variably conserved (60–100% identity) regions among them (from a BLASTN comparison with e-value ≤ 10^-4^). Predicted glycosyltransferases are colored in orange, *wzy* and *gnd* homologs in yellow and purple, respectively. N.T., new K-type; N.D., K-type not determined.

The *cps*_Kp13_ has a genomic organization similar to other *K. pneumoniae cps* clusters, and it can be divided into three regions as shown in Figure 1. The 5’ end or region 1 (from *gal*F to *wba*P) contains conserved genes responsible for polymer assembly and translocation [12]. The central region or region 2 contains genes encoding serotype-specific GTs and *gnd*. The 3’ end or region 3 is more variable among different capsular types, with some containing the *man*CB operon that encodes GDP-D-mannose, like serotypes K1 and K5 [15]. Similarly to serotypes K9 and K52, the 3’ end of the *cps*_Kp13_ gene cluster contains the *rml*BADC operon for the synthesis of dTDP-L-rhamnose instead of the *man*CB operon [15]. The genes *wzx* and *wzy* are also found in the 3’ region of the Kp13 *cps* cluster. This region is succeeded by defective IS elements and a prophage fragment (Figure 1). The discussed conservation of region 1 and variability of region 2 can be readily observable on a comparison of the *cps* loci of different K-types deposited in NCBI (Figure 2).

### The *cps*_Kp13_ monosaccharide biosynthesis pathways: UDP-D-glucuronate, UDP-D-galacturonate and L-rhamnose

**Figure 3:**
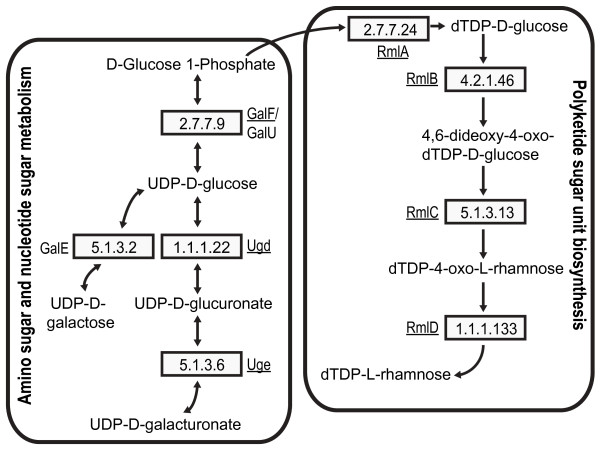
**Amino- and polyketide sugar production in *K. pneumoniae* Kp13.** Pathways leading to UDP-D-galacturonate, UDP-D-galactose and dTDP-L-rhamnose are shown, as these residues could be present in the capsular structure of Kp13. Enzymes coded by genes present in the *cps*_Kp13_ cluster are underlined.

As in other bacteria that produce group-1 capsules, *gal*F delimits the 5’ region of *cps*_Kp13_. This gene shows 100% identity to the *gal*F sequence present in *K. pneumoniae* NK8 [GenBank:BAI43699], which codes for a UTP-glucose-1-phosphate uridylyltransferase (EC 2.7.7.9, Figure 3). This enzyme belongs to the nucleotidyltransferase family and catalyzes the reaction UTP + α-D-glucose 1-phosphate ↔ diphosphate + UDP-D-glucose. This enzyme is important because UDP-D-glucose serves as a precursor for the biosynthesis of bacterial lipopolysaccharides and capsular polysaccharides. It is also possible that the *gal*F product interacts with the product of *gal*U, thus elevating UDP-D-glucose concentration in the cell and providing more material for the synthesis of capsular polysaccharides [11]. In fact, a *gal*U homolog found in Kp13 outside the *cps* region (KP04702) shows 94% identity (BLASTP) to GalU from *Shigella flexneri* [Swiss-Prot:P0AEP6]. Immediately downstream of the *rml*BADC operon, the gene *ugd* is found (Figure 1). It encodes a UDP-glucose 6-dehydrogenase (EC 1.1.1.22). As depicted in Figure 3, this enzyme converts UDP-D-glucose to UDP-D-glucuronate, a common constituent of bacterial capsules [7]. As with other sequences located in the 3’ region of the *cps*_Kp13_ gene cluster, this coding sequence exhibits remarkable amino acid conservation. It is 100% identical to Ugd from *K. pneumoniae* strains NK8 [GenBank:BAI43716] and VGH404 serotype K5 [GenBank:BAI43755] (Table 1), both studied by Shu et al. [15]. Uge catalyzes the conversion of UDP-D-glucuronate to UDP-D-galacturonate (Figure 3), which is also present in both bacterial capsules and LPS. In fact, Kp13 has two copies of this gene, *uge*-1 (KP03793) and *uge*-2 (KP03786). A NAD-dependent epimerase domain (Pfam accession no. PF01370) is predicted to occupy amino acids 4 to 230 in both Uge sequences. Two copies of *uge* are also found in the genome of *K. pneumoniae* subsp. *rhinoscleromatis* (which produces a K3 capsule), one in the *cps* cluster and an inverted adjacent copy in the cluster for LPS synthesis [16]. As the K3 CPS contains D-galacturonate in its composition, *uge* was considered the last gene of its *cps* cluster [16] instead of *ugd* as usually regarded [15,17]. In Kp13 *uge*-1 should also be considered within the *cps* since the genes necessary to the flippase and polymerase activities, *wzx* and *wzy* respectively, are located downstream (Figure 1); therefore, D-galacturonate could also form the Kp13 CPS composition. The effects of a *uge* null mutation on colonization and virulence were studied in *K. pneumoniae* 52145, which is a highly virulent strain able to colonize different surfaces [18]. A *uge* deletion reduced colonization and rendered the strain completely avirulent in an experimental model of pneumonia [18]. This suggests that the *uge*-1 and/or *uge*-2 mutation in Kp13 could have important, measurable effects on colonization and virulence.

In the *cps*_Kp13_ cluster, genes encoding enzymes that participate on the synthesis of dTDP-L-rhamnose from glucose 1-phosphate are found immediately downstream of the *gnd* gene (Figure 1). The *rml*BADC genes were found in three capsular serotypes studied by Shu et al. [15]: K9, K14 and K52. In serotypes K9 and K52, these genes are also found downstream of *gnd*. The lengths of the products encoded by *rml*A, *rml*B, *rml*C and *rml*D are shown in Table 1, along with the best BLAST hits for these genes. The gene *rml*A codes for a glucose-1-phosphate thymidylyltransferase (EC 2.7.7.24), which catalyzes the first reaction of L-rhamnose synthesis: dTTP + α-D-glucose 1-phosphate → diphosphate + dTDP-D-glucose (Figure 3). The second reaction is performed by dTDP-D-glucose 4,6-dehydratase (EC 4.2.1.46, Figure 3), the product of *rml*B, which catalyzes the dehydration of dTDP-D-glucose to dTDP-4-keto 6-deoxy-D-glucose. Epimerization at the C3’ and C5’ positions of this molecule is performed by dTDP-4-dehydrorhamnose 3,5-epimerase (*rml*C, EC 5.1.3.13, Figure 3), producing dTDP-4-oxo-L-rhamnose. Finally, dTDP-4-dehydrorhamnose reductase (EC 1.1.1.133, Figure 3), encoded by *rml*D, catalyzes the reduction of dTDP-4-oxo-L-rhamnose to dTDP-L-rhamnose, which can be subsequently linked to the capsular polymer by a specific rhamnosyltransferase. All three conserved regions (the Y-X_3_-K loop, the Wierenga motif G-X_2_-G-X_2_-G and the STDYVF sequence) discussed by Giraud and Naismith [19] are present in Kp13’s RmlD.

Whereas the chemical composition of the Kp13 capsule remains to be determined, the pyrosequencing-based genomic analysis of *cps*_Kp13_ allowed the identification of sugar metabolic pathways. Genes encoding enzymes for the biosynthesis of sugar nucleotide precursors in the Kp13 capsule, such as UDP-D-glucose, UDP-D-glucuronate, UDP-D-galacturonate and dTDP-L-rhamnose, are found in the *cps* cluster. Thus, the capsule of Kp13 may contain any of these sugar nucleotide precursors. In particular, the presence of two genes in the *cps* cluster encoding possible rhamnosyltransferases suggests that L-rhamnose makes up part of the Kp13 capsule (see discussion below). On the other hand, *gal*E (KP02995) was identified outside the *cps* region, and it encodes a UDP-glucose 4-epimerase with roles in the amino sugar and nucleotide sugar pathways producing UDP-D-galactose from UDP-D-glucose (Figure 3). The presence of this gene suggests that the capsule composition of Kp13 could also include UDP-D-galactose derivatives. Neither the *man*A, *man*B and *man*C genes of the *cps* cluster nor other genes of the mannose and fucose biosynthesis pathways were identified in the Kp13 genome. This suggests that the CPS of Kp13 does not contain GDP-D-mannose or GDP-L-fucose derivatives.

### Proteins involved in translocation, surface assembly and polymerization: Wzi, Wza, Wzb, Wzc, Wzx and Wzy

The deduced amino acid sequences of the *wzi* and *wza* genes found in *cps*_Kp13_ show 98% and 97% identity, respectively, with homologs from *K. pneumoniae* VGH484 (Table 1), and both proteins were predicted to localize in the outer membrane (PSORTb scores: Wzi, 9.52; Wza, 9.92). Moreover, a signal peptide was predicted for the *wzi* gene product. Analysis of the secondary structure of the Kp13 Wzi protein using PSIPRED showed that it is rich in β-sheet regions (data not shown), an observation that has been experimentally confirmed for a Wzi homolog in *E. coli* [GenBank:AAD21561.1] [20] which shares 98% identity with that of Kp13. Also, Rahn et al. [20] established the importance of the Wzi outer membrane protein for capsule synthesis by showing that *wzi* mutants have lower amounts of cell-associated capsular polysaccharide.

The *wza* product of Kp13 has 92% identity with Wza from *E. coli* [GenBank:AAD21562.1], which has been shown to be an integral lipoprotein with exposed regions on the cell surface. The *E. coli* protein forms a ring-like structure responsible for polymer translocation through the outer membrane [12]. Wzc and Wzb are a tyrosine autokinase and its cognate acid phosphatase, respectively, and they are ubiquitously found in group 1 capsule clusters [12,21]. The Kp13 Wzc protein was predicted to have two transmembrane regions, like its counterpart in the *K. pneumoniae* strain Chedid, with which it shares 72% amino acid identity [Swiss-Prot:Q48452]. The inner membrane is the probable location of Kp13’ Wzc (PSORTb score 9.99), in agreement with its role in capsule synthesis. Wzc is involved in the translocation of capsular polysaccharide from the periplasm to the cellular surface through formation of a complex with Wza [22]. Wzc undergoes autophosphorylation of its tyrosine-rich C-terminal residues (of the last 17 residues in Kp13 Wzc, eight are Tyr) potentially modulating the opening and closing of the translocation channel [12]. The Wzb protein (EC 3.1.3.48) of Kp13 is probably located in the cytoplasm (PSORTb score: 9.26). Wzb catalyzes the removal of a phosphate group from phosphorylated Wzc and is necessary for continued polymerization of the repeat units [12]. Sequence conservation of the Wzy and Wzx proteins is usually low [23]. The localization of *wzx* and *wzy* in Kp13 is different from that observed in various K-serotypes by Shu et al. [15], in which the genes usually mapped upstream of *gnd*. In Kp13, both genes are located downstream of *gnd*, in region 3 of the *cps* cluster, and *wzy* is transcribed in the opposite direction relative to other *cps* genes. Wzx is an inner membrane protein that transfers the polysaccharide units, assembled in the cytoplasm, into the periplasm, thus acting as a flippase [12]. The Wzx protein from *cps*_Kp13_ has 10 predicted transmembrane segments and is 411 aa long, which is in agreement with a previous study of this protein in *E. coli* that predicted 10–12 transmembrane segments [23]. BLASTP against the NCBI database shows that the best hit (64% identity) is a putative Wzx protein from *E. coli* TA271 (NCBI accession no. ZP_07523140, Table 1). A polysaccharide biosynthesis domain (Pfam accession no. PF01943), common to Wzx proteins, was found spanning amino acids 8 to 275 of Kp13 Wzx.

Wzy from Kp13 is 348 aa long and also had 10 predicted transmembrane segments, similar to the Wzy proteins of other *Enterobacteriaceae* that have 10–11 transmembrane segments [24]. This protein is believed to be a polysaccharide polymerase, although experimental evidence for this activity has not yet been reported due to the technical difficulty of working with Wzy in vitro [12]. NCBI BLASTP searches show that the best hit (35% identity) for Wzy is a conserved protein from *Thermoanaerobacter wiegelii* [GenBank:ACF14522.1] (Table 1).

It is remarkable that the *wzy* gene from isolate Kp13 is transcribed in the opposite direction compared to other genes of the *cps* cluster, a characteristic that to our knowledge has not been reported for previously studied *cps* clusters, as can be observed in Figure 2, where the position of *wzy* within different *K. pneumoniae cps* loci is highlighted.

Downstream *wzy*, we have identified an 862-bp region showing 70% identity to an IS element of the IS3 family [GenBank:CP002438.1]. No terminal inverted repeats or target site duplications were found in this element. Although three ORFs identified within this putative IS showed significant identity to distinct transposases, these structures do not seem to encode functional enzymes. The occurrence of mutations leading to premature stop codons and/or frameshifts might have rendered this transposase non-functional. Alternatively, this chimeric structure could have resulted from homologous recombination events with other transposase-encoding genes. Upstream *wzy*, there is a 1539-bp ORF whose deduced amino acid sequence shows 31% identity to a defective tail fiber protein of a Mu-like prophage identified in *Dickeya dadantii* [GenBank:ADM97620]. Notably, other prophage genes were absent. The location of *wzy* between two defective mobile genetic elements suggests that this gene may have been incorporated into Kp13’s *cps* via an ancient horizontal gene transfer event. Subsequently, these mobile genetic elements may have been truncated in order to assure that *wzy* would be permanently present in the *cps*_Kp13_ region, which is desirable because capsule assembly is a Wzy-dependent process.

The conserved gene *gnd*, found in the central region of *cps*_Kp13_, encodes a 468 aa protein (6-phosphogluconate dehydrogenase, EC 1.1.1.44, Figure 3) that catalyzes the conversion of 6-phospho-D-gluconate to D-ribulose 5-phosphate during the third step of the pentose phosphate pathway. This gene was found in all of the *cps* gene clusters studied by Shu et al. [15] and shows a high degree of conservation among them, which would be expected from an evolutionary standpoint due to the central role of this metabolic pathway. At the protein sequence level, the best hit (99% identity) for Kp13’s *gnd* product is an ortholog from strain VGH484, serotype K9 [GenBank:BAI43786.1] (Table 1).

### Kp13’s *cps* gene cluster has five GTs: WbaP, Orf8, Orf9, Orf10 and Orf19

The products of *wba*P, *orf*8, *orf*9, *orf*10 and *orf*19 are GTs, enzymes specialized on the polymerization of sugar molecules into existing molecules, which can be carbohydrates, lipids or proteins. Because of the variety of modifications catalyzed by GTs it is difficult, based on sequence analysis alone, to define the exact outcome of each reaction [25], even though they may play an important part on the diversity of capsular structures encountered in *K. pneumoniae*. The number of GTs in *K. pneumoniae*’s *cps* cluster is variable, ranging from three (serotypes K1 and K2) to six as reported by Shu et al. [15]. Kp13 has a total of five GTs, four of these located contiguously (*wba*P, *orf*8, *orf*9 and *orf*10) and one of them found on the 3’ end of the cluster (*orf*19). All the GTs found on Kp13’s *cps* gene cluster have been predicted to belong to the family 2 GTs, comprising enzymes that use an inverting catalytic mechanism which modifies the anomeric configuration of the transferred sugar [26]. *wba*P (formerly *rfb*P) is the first GT on Kp13’s *cps* gene cluster and encodes a 482 aa long UDP-Gal::undecaprenolphosphate Gal-1-P transferase, which catalyzes the initial transfer of galactose-1-phosphate to an undecaprenol phosphate acceptor, thus initiating the capsule polymer synthesis. This protein was predicted to be located in the cytoplasmic membrane (PSORTb score: 10.0) and may contain five transmembrane-spanning regions. A conserved WbaP phosphotransferase domain (IPR017472, e-value 7.5e-194) is also found ranging from amino acids 21 to 482. NCBI BLASTP searches showed identity of up to 80% with WbaP from other *K. pneumoniae* and *E. coli*. The protein presents two conserved DxD motifs, which are widespread in GTs and are thought to be involved in metal/nucleotide binding and catalysis [27,28]: DED, ranging from amino acids 356–358 and DVD, 442–444 aa. The latter has been found in all but one of 12 different capsular serotypes studied by Shu et al. [15]. *orf*8 (KP03804) encodes a 302 aa uncharacterized GT whose amino acid sequence shows 67% identity (Table 1) with putative rhamnosyltransferases from *K. pneumoniae* strain A1517 showing a unique capsular serotype [GenBank:BAF75773.1] [14]. The GT encoded by *orf*9 (KP03803) is predicted to be 298 aa long, with a best hit on NCBI BLASTP with a putative dTDP-rhamnosyltransferase from *D. dadantii* [GenBank:ADM97617.1] (63% identity, Table 1). *D. dadantii* is a distantly related plant pathogen of the *Enterobacteriaceae* family. Interestingly, there is little similarity between *orf*9 and other *K. pneumoniae* sequences. The highest identity match (31%) is with a putative rhamnosyltransferase from strain VGH484 [GenBank:BAI43783.1]. The presence of the *rml*BADC genes (previously discussed) together with the possible rhamnosyltransferases provides appealing evidence that L-rhamnose makes part of Kp13’s capsular structure. *orf*10, the third gene encoding a putative GT located in region 2 of the Kp13 *cps* cluster, is predicted to code for a 253 aa long protein with a conserved domain of unknown function spanning amino acids 36 to 193 (Pfam accession no. PF04765). As with *orf*9, the best hit (57% identity, Table 1) is also with a sequence encoding a putative GT from *D. dadantii* [GenBank:ADM97619.1]. There was no similarity between the *orf*10 (KP03802) product and other published *Klebsiella* sequences.

Finally, the last GT from *cps*_Kp13_, termed *orf*19, is located on the 3’ end of the *cps* cluster and encodes a predicted 330 aa product. This protein has similarity with several uncharacterized GTs family 2 from different *Enterobacteriaceae*, including *E. coli* TA271 [GenBank:EGI36158.1] (58% identity), *D. dadantii* [GenBank:ADM97622.1] (38%) and *Cronobacter sakazakii* [GenBank:ABX51890.1] (34%). Only a general domain of the GTs family 2 was found in this protein, spanning amino acids 7 to 145 (Pfam accession no. PF00535).

### In silico serotyping

Using molecular serotyping for the *cps* cluster, Brisse et al. [29] showed that very distinct PCR-RFLP patterns (C patterns) were obtained for most of the K serotypes, indicating that differences in antigenic specificity among serotypes are due to differences in *cps* gene content. Thus, we have also applied in silico molecular serotyping to determine the capsular serotype of isolate Kp13. For this approach, the sequence between the primers published by Brisse et al. [29] was used to search in silico for restriction sites of the HincII endonuclease. This sequence spanned 12,031 bp from *wzi* to *gnd*, and the in silico restriction analysis identified 12 restriction sites, corresponding to 11 restriction fragments (Table 2). The fragments, ranging in size from 368 to 1,777 bp, were selected for analysis as suggested by Brisse et al. [29] (Table 2). The *cps*_Kp13_ RFLP pattern was compared to 102 previously published C patterns [29]. None of the reference patterns matched the one displayed by Kp13 (see Additional file 1). The similarity score for Kp13 was greater than 10.4 (MST cutoff value score ≥ 0.75), thus providing additional evidence that Kp13 K-type is a new serotype.

**Table 2:** **In silico HincII restriction pattern obtained for the 12,031 bp sequence spanning *wzi* to *gnd* in the Kp13 *cps* gene cluster**

**Start**	**End**	**Cut site**	Restriction fragment size between adjacent sites* (bp)
548	553	550	
1,561	1,566	1,563	1,013
1,638	1,643	1,640	77
2,458	2,463	2,460	820
2,550	2,555	2,552	92
7,129	7,134	7,131	4,579
7,260	7,265	7,262	131
7,266	7,271	7,268	6
7,634	7,639	7,636	368
9,411	9,416	9,413	1,777
10,798	10,803	10,800	1,387
10,863	10,868	10,865	65

* Fragments used for this analysis are underlined.

### In vitro K-serotyping

Kp13 showed a weak positive reaction with both K9 and K34 antisera that could not be resolved by modifying antiserum dilution or quellung reaction. This result is not surprising since cross-reactions with the type-specific antisera is commonly observed among *K. pneumoniae* clinical isolates due to the activity of common genetic elements among distinct *cps* clusters [30]. In fact, the *rml*BADC genes are also present in the *cps* cluster displayed by serotype K9 [15], and its CPS is composed of D-glucuronate, D-galactose and L-rhamnose residues [31]. Given the gene content of *cps*_Kp13_ and the presence of *gal*E on the Kp13 genome, these residues could all be synthesized by this isolate, hence cross-reactions were not unexpected. From the comparison of *cps*_Kp13_ and *cps*_VGH484_ (K9, Figure 2) it is clear that they have common genes, but the Kp13 *cps* also has distinguishing features like its repertoire of GTs, the presence of *uge*-1 and a different cluster organization (e.g. the positions of *wzy* and *wzx*). In the same line of evidence, the CPS of serotype K34 is composed of L-rhamnose, D-glucose and D-galacturonate residues [32], all of which also potentially present in the Kp13 CPS as discussed earlier, and D-galacturonate being produced by the epimerase activity from the *uge*-1 product. No *cps* sequences from K34 isolates were found on public databases. Nevertheless, our results indicate that Kp13 possess a unique serotype since it showed a distinct RFLP pattern compared to those 102 patterns, including representatives of serotypes K9 and K34, previously described [29]. It has also been observed that *cps*-PCR genotyping seems to be a more sensitive and specific way for detecting novel serotypes [14], and our pyrosequencing-based approach together with the careful scrutinization of each CDS in the cluster and the in vitro results supports the finding that Kp13 synthesizes a novel CPS.

### Regulation of *cps* gene expression in Kp13

**Figure 4:**
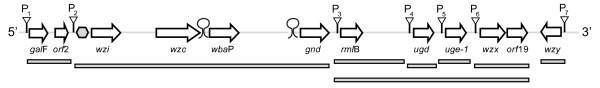
**Model of regulation in the *K. pneumoniae* Kp13 *cps* cluster.** Only selected genes are shown. The promoters are depicted as upside-down triangles, and the JUMPStart element is shown as a hexagon. The rectangles under each cluster represent transcriptional units, and the stems are possible Rho-independent attenuators. P3 could either drive the transcription of *rml*B through *orf*19 or there could be other promoters (P4, P5 or P6). The possible transcriptional units are depicted.

The transcriptional regulation of *cps* genes is thought to be under the control of three promoters, P1, P2 and P3, which are located upstream of *gal*F, *wzi* and *rml*B, respectively [13,15]. As previously shown for other strains by Shu et al. [15], in the *cps*_Kp13_ cluster the transcripts driven by P1 and P2 should consist of *gal*F/*orf*2 and *wzi* to *gnd*, respectively (Figure 4). Regulatory elements have been identified within the promoters P1 and P2 of the *cps*_Kp13_ cluster. Promoter P1 contains the regulatory RcsAB box operator (5-TAAGATTATTCTCA-3’) that is essential for the induction of Rcs-regulated promoters by way of its interaction with the regulators RcsB and RcsA [33]. Predicted *rcs*B and *rcs*A genes are present in the Kp13 genome, encoded, respectively, by predicted coding sequences KP00953 and KP04844.

The JUMPStart element was found within promoter P2 (Figure 4). This element was identified upstream of a number of bacterial *cps* clusters [15,34]. The 8-bp *ops* element (5’-GGCGGTAG-3’) is located within JUMPStart and has been reported to function as a binding site for the RfaH activator protein [35]. Indeed, *rfa*H is found elsewhere in the Kp13 genome (KP31625), and its deduced amino acid sequence displays 80% identity with an ortholog from *E. coli* K12 [Swiss-Prot:P0AFW0]. A possible stem-loop structure (Figure 4) related to the Rho-independent transcription attenuator is located in the intergenic region between *wzc* and *wba*P of the *cps*_Kp13_ cluster, as predicted by the ARNold web server [36] with a calculated free energy of −8.49 kcal/mol. Similar features have also been identified in other *cps* clusters from *K. pneumoniae*[9,15]. Additionally, a second putative stem-loop structure (Figure 4) was predicted downstream of *orf*10 (ΔG = −8.20 kcal/mol). Further studies are necessary to confirm the implications of this finding; a stem-loop in this position has not been previously described. The transcription of *cps*_Kp13_ region 3 may occur from different promoters. For instance, the P3 promoter upstream *rml*B may transcribe a polycistronic mRNA from this gene up to *orf*19 or, alternatively, each individual promoter predicted in this region may drive the transcription of a limited number of genes (Figure 4).

Notably, *wzy* is located between defective mobile elements and is transcribed in the opposite direction of other genes in the *cps* cluster (Figure 1). Thus, it should have its own promoter (possibly P7). A putative −10 box was found, separated by 15 bp from its −35 counterpart, but no obvious RBS could be identified. This observation raises the question of how Kp13 coordinates expression of *wzy*, since this protein is also essential for the formation of CPS.

Deviations from the −10 and −35 consensus sequences significantly modify the strength of each promoter [37], so the number of promoters could in fact be different from that proposed here. Still, their roles in *cps* cluster regulation deserve further experimental study, and our sequence-based bioinformatic approach provides candidates for follow-up.

## Conclusions

In this study, we report a unique *cps* cluster organization in Kp13, a multidrug-resistant, KPC-producing *K. pneumoniae* strain that caused a large outbreak in a Brazilian teaching hospital. The Kp13 *cps* cluster contains all of the genes necessary for capsule biosynthesis. Based on the sugar metabolic pathways identified in *cps*_Kp13_ and in other genomic regions, we have predicted that the capsule composition of Kp13 may include D-glucose, D-glucuronate, D-galacturonate, D-galactose and L-rhamnose residues.

## Methods

### Ethics statement

This study was approved by the Ethics Committee of the Universidade Estadual de Londrina (UEL) under reference number CAAE: 3356.0.000.268-09. Clinical assessment and blood sampling were performed after diagnostic routine procedures in the intensive care unit of the Hospital Universitário-UEL, with written informed consent of the patient.

### Bacterial strain

Between February and May 2009, a teaching hospital located in Southern Brazil experienced its first outbreak of nosocomial infections due to KPC-producing *K. pneumoniae*. The KPC-producing *K. pneumoniae* isolate Kp13 was recovered from the blood culture of a patient admitted to the intensive care unit with diabetes mellitus and cranial encephalic trauma. Automated bacterial identification was conducted with a MicroScan WalkAway apparatus (Dade Behring, Sacramento, CA, USA). Kp13 was phenotypically detected as a carbapenemase producer by the modified Hodge [38], and the specific *bla*_KPC-2_ gene was identified by PCR and amplicon sequencing using previously described primers and cycling conditions [39].

Kp13 was identified as *K. pneumoniae* subsp. *pneumoniae* by showing that its *rpo*B gene has 99% identity to *rpo*B of *K. pneumoniae* subsp. *pneumoniae* strain MGH 78578 [GenBank:ABR79724.1].

### DNA sequencing, assembly and sequence analysis

Genome sequencing of Kp13 was performed at the Unidade Genômica Computacional - UGC/LNCC Facility (http://www.labinfo.lncc.br/index.php/ugc) located in Petrópolis, Rio de Janeiro, Brazil, using the Genome FLX sequencer (454 Life Science/Roche). Both shotgun and 3 kb paired-end libraries were constructed, and sequencing was carried out using FLX-Titanium chemistry. A paired-end (PE) library analysis was applied to determine the orientation and relative position of contigs produced by de novo shotgun sequencing. The data consisted of a total of 1,336,815 whole-genome shotgun reads and 558,997 paired-end reads.

Assembly of the sequence data into contigs and scaffolds was performed using the GS De Novo Assembler software provided by 454 Life Sciences/Roche (v 2.5). The high-quality reads were assembled into 151 contigs and 15 scaffolds, comprising 5.9 Mb of sequence. For the *cps*_Kp13_ region from *gal*F to *wzy*, 99.9% of the bases had Phred-like quality ≥ 60. The SABIA annotation pipeline [40] was used to predict protein-coding genes and non-coding RNA genes. With the aim of detecting complete *cps*_Kp13_ genes, functional annotation of the ORFs was performed by searching the NCBI non-redundant protein database using BLASTX, followed by manual curation.

Protein subcellular localizations and signal peptides were predicted using PSORTb 3.0 [41] with default parameters for Gram-negative bacteria. A score of 7.5 was considered to be the cutoff for identification of protein localization. Transmembrane regions were analyzed using TMHMM [42]. Protein secondary structures were predicted using the PSIPRED web server [43], available at http://bioinf.cs.ucl.ac.uk/psipred. Prediction of promoters was performed using the in-house SABIA platform as well as the BPROM program (http://linux1.softberry.com), which searches for promoters under the control of the sigma factor 70. Ribosome binding sites search was performed using the RBS finder software that is included in the SABIA platform. EasyFig [44] was used to generate the structural comparison of *cps*_Kp13_ and other sequenced *cps* loci.

### In silico serotyping

An in silico serotyping approach was applied using the Molecular Serotyping Tool (MST) [45]. MST is a program for computer-assisted molecular identification of restriction fragment length polymorphisms (RFLP) patterns, in which the concepts of similarity and alignment between RFLP patterns were adapted from Needleman and Wunsch's dynamic programming algorithm. By analogy, RFLP patterns represented by ordered fragment sizes can be aligned, and their similarity can be calculated as the sum of penalties for edit operations (insertions, deletions or substitutions) that transform one pattern into another [45]. MST, available at http://www.cebio.org/mst, was originally designed for the identification of RFLP patterns from *Escherichia coli* and the *Shigella* O-antigen gene clusters [46,47]. At present, identification of *K. pneumoniae* serotypes can also be achieved because the RFLP patterns of the amplified capsular antigen gene clusters of all known *Klebsiella* serotypes were published by Brisse et al. [29].The RFLP of Kp13 was determined and compared to those already described. All scores were used to build a distance matrix in a PHYLIP compatible format [48]. The distance matrix was used to reconstruct a phylogeny by the UPGMA method with the NEIGHBOR program, available in the PHYLIP package. The tree generated by UPGMA was visualized with the graphical viewer FIGTREE (http://tree.bio.ed.ac.uk/software/figtree/). To improve the analysis of the UPGMA tree, the two-time-scales were applied. The MST distance cutoff that is able to distinguish between two serotypes is 1.5, and the scale-adjusted measure should be interpreted as 0.75.

### In vitro K-serotyping

Isolate Kp13 was sent to the International *Escherichia* and *Klebsiella* Reference Center (WHO), Statens Serum Institut, Copenhagen, Denmark, for serotyping. Briefly, K-typing was done by counter-current immunoelectrophoresis (CCIE) against antiserum pools as previously described [49]. Then, Kp13 was tested against with the specific K-antisera of the reacting pool. The presence of a visible capsule by wet-mount microscopy with Indian Ink, quellung reaction, was also carried out with specific antisera since a cross-reaction had occurred.

### Nucleotide sequence accession numbers

The *cps*_Kp13_ sequence and annotations are available from Genbank (http://www.ncbi.nlm.nih.gov/Genbank) under accession number [GenBank:JN377737]. The GenBank accession numbers for other sequences discussed in the manuscript are [GenBank:JN377738] (*gal*E), [GenBank:JN377739] (*gal*U), [GenBank:JN377740] (*rfa*H), [GenBank:JN377741] (*rcs*B) and [GenBank:JN377742] (*rcs*A).

## Competing interests

The authors declare that there are no competing interests.

## Authors’ contributions

ECV and MP provided the Kp13 isolate and performed bacterial identification. ALG, MFN and ATRV conceived the pyrosequencing strategy. Annotation and bioinformatics analyses were performed by LGPA, LFGZ, PIPR, RCP, ACG and MFN. The manuscript was prepared by PIPR, RCP, ACG and MFN. All authors read and approved the final manuscript.

## Supplementary Material

Additional file 1 **Cluster analysis of 103 RFLP patterns after MST analysis.** MST distances between serotypes are represented as alignment scores, with 0.75 used as the scale-adjusted threshold for distinguishing two serotypes. *K. pneumoniae* Kp13 is labeled as KP13, while the other serotypes follow the C-pattern nomenclature from Brisse et al. [29]. Click here for file
