# Supplementary figures and images for: Pyrosequencing-based analysis reveals a novel capsular gene cluster in a KPC-producing Klebsiella pneumoniae clinical isolate identified in Brazil
Source: BMC Microbiol. 2012 Aug 11;12:173. doi: 10.1186/1471-2180-12-173 (PMC3438125; doi:10.1186/1471-2180-12-173)

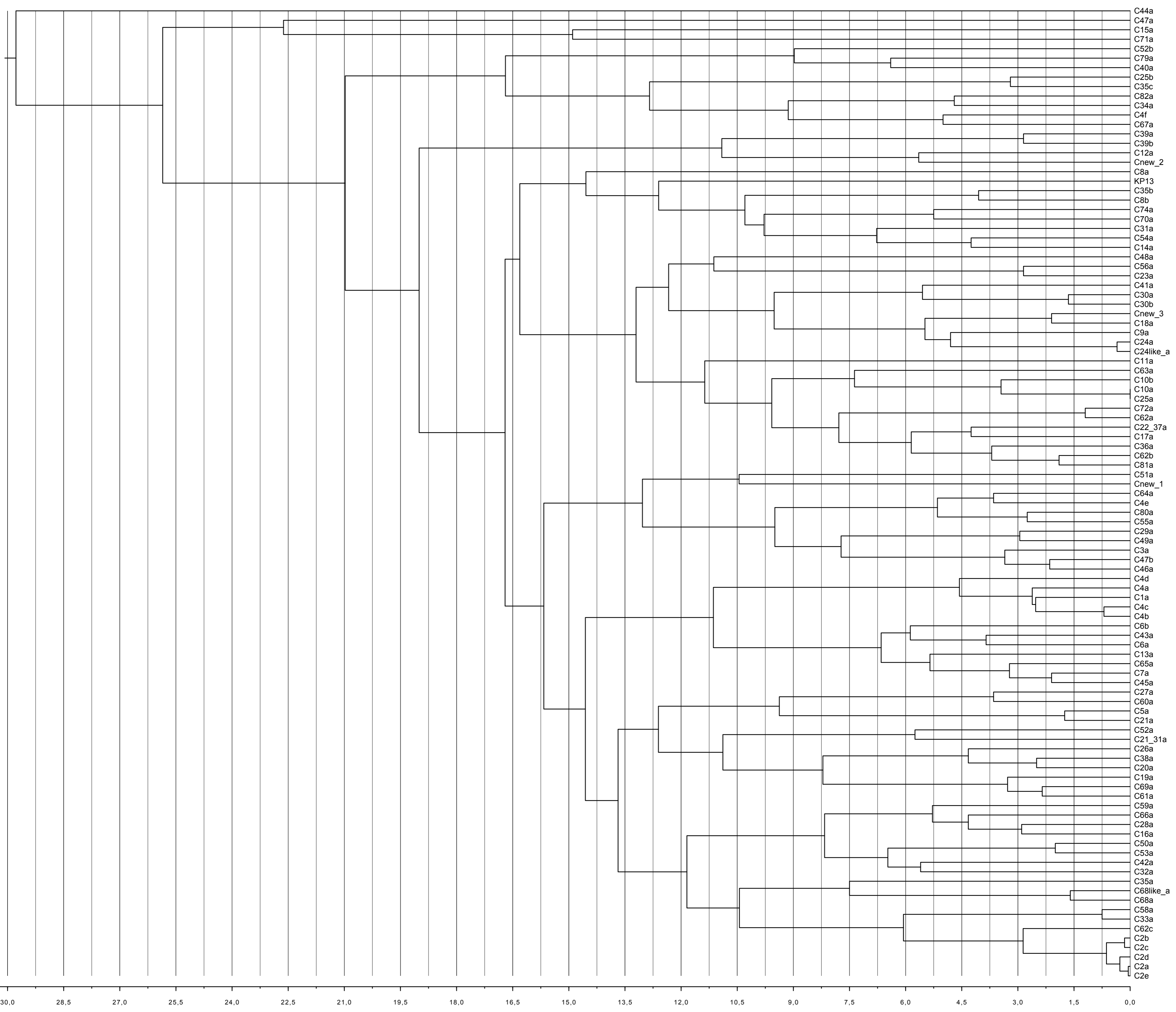

Supplement: Additional file 1 — Cluster analysis of 103 RFLP patterns after MST analysis. MST distances between serotypes are represented as alignment scores, with 0.75 used as the scale-adjusted threshold for distinguishing two serotypes. K. pneumoniae Kp13 is labeled as KP13, while the other serotypes follow the C-pattern nomenclature from Brisse et al. [29]. [file 1471-2180-12-173-S1.pdf]
